# Distribution and Relationships of Polycyclic Aromatic Hydrocarbons (PAHs) in Soils and Plants near Major Lakes in Eastern China

**DOI:** 10.3390/toxics10100577

**Published:** 2022-09-30

**Authors:** Zhiwei Zhao, Wei He, Ruilin Wu, Fuliu Xu

**Affiliations:** 1 MOE Laboratory for Earth Surface Processes, College of Urban and Environmental Sciences, Peking University, Beijing 100871, China; 2 Beijing Key Laboratory of Water Resources & Environmental Engineering, China University of Geosciences (Beijing), Beijing100083, China

**Keywords:** PAHs, distribution, correlation, soil, plants, Eastern China

## Abstract

The distributions and correlations among polycyclic aromatic hydrocarbons (PAHs) in soils and plants were analyzed. In this study, 9 soil samples and 44 plant samples were collected near major lakes (Hongze Lake, Luoma Lake, Chaohu, Changhu, Danjiangkou Reservoir, Wuhan East Lake, Longgan Lake, Qiandao Lake and Liangzi Lake) in eastern China. The following results were obtained: The total contents of PAHs in soil varied from 99.17 to 552.10 ng/g with an average of 190.35 ng/g, and the total contents of PAHs in plants varied from 122.93 to 743.44 ng/g, with an average of 274.66 ng/g. The PAHs in soil were dominated by medium- and low-molecular-weight PAHs, while the PAHs in plants were dominated by low-molecular-weight PAHs. The proportion of high-molecular-weight PAHs was the lowest in both soil and plants. Diagnostic ratios and principal component analysis (PCA) identified combustion as the main source of PAHs in soil and plants. The plant PAH monomer content was negatively correlated with Koa. Acenaphthylene, anthracene, benzo[k]fluoranthene, benzo[b]fluoranthene and dibenzo[a,h]anthracene were significantly correlated in plants and soil. In addition, no significant correlation between the total contents of the 16 PAHs and the content of high-, medium-, and low-molecular-weight PAHs in plants and soil was found. *Bidens pilosa* L. and *Gaillardia pulchella* Foug in the Compositae family and cron in the Poaceae family showed relatively stronger accumulation of PAHs, indicating their potential for phytoremediation.

## 1. Introduction

Polycyclic aromatic hydrocarbons (PAHs) are a class of organic compounds containing two or more benzene rings connected by fused or non-fused rings. PAHs are released into the environment from both natural and anthropogenic sources. The incomplete combustion of organic matter from human activities, such as the combustion of coal, firewood, and other biomass, as well as exhaust emissions from diesel and gasoline vehicles, can lead to the production of PAHs [1]. Natural activities, such as forest fires, grassland fires and volcanic eruptions also produce PAHs [2,3,4]. PAHs are carcinogenic and mutagenic [5,6]. They are ubiquitous in water, the atmosphere, soil, and sediment. They may harm human health if they enter the body through exposure to contaminated environments or the food chain [7,8].

In terrestrial environments, soil is the primary environmental repository for semi-volatile organic compounds, such as PAHs [9]. PAHs in soil can be derived from both dry and wet deposition of PAHs from the atmosphere. Wastewater discharge and irrigation may also increase the concentration of PAHs in soil [10]. For PAH-contaminated soil remediation, phytoremediation is one of the attractive strategies due to its environmental friendliness and relatively low cost [11]. Plants can absorb and accumulate PAHs from contaminated soil [12]. In addition to soil, plants can also absorb PAHs directly from the atmosphere because of the lipophilicity of both PAHs and the plant cuticle [13]. Contaminated particulates deposited on leaves are another source of PAHs in plants [14]. Studies have shown that the differences in PAHs in plant samples reflect spatial differences in PAHs in the atmosphere [15].

There have been a variety of studies on PAHs in soils around the world. The content and distribution of PAHs in soils from different countries have been reported [16,17,18]. Some studies focus on the sources, health risks and ecological risks of PAHs in soil [19,20]. Studies on the remediation of PAH-contaminated soils have also been conducted [21,22].

Compared to soil studies, there are fewer studies on PAHs in plants. The type of plant species is one of the factors that affect the concentration of PAHs in plants [23,24]. Several plants have been found to have the potential to remediate PAH-contaminated soils [25,26], but the distribution of PAHs in the plant-soil system has not been discussed.

Various studies have been conducted on PAHs in soil [27,28,29], and several studies have measured the contents of PAHs in plants. However, few studies have focused on soil-plant systems. In this study, soil samples and plant samples near major lakes in eastern China were collected to: (1) investigate the contents and distribution of 16 PAHs in soil and plants; (2) preliminarily identify the emission sources of PAHs; (3) reveal the distribution of PAHs in the soil-plant system and the possible relationships between PAHs in soil and plants; (4) explore the possibility of phytoremediation in PAH-contaminated soil.

## 2. Materials and Methods

### 2.1. Sample Collection

The nearshore areas of nine lakes in two major lake areas in eastern China were selected for this study. The nine lakes include Hongze Lake and Luoma Lake in the Huaihe River Basin, and Chaohu, Changhu, Danjiangkou Reservoir, Wuhan East Lake, Longgan Lake, Qiandao Lake, and Liangzi Lake in the middle and lower reaches of the Yangtze River. Detailed information on the nine lakes is provided in Table S1.

Soil samples (n = 9) were collected from nearshore areas of the lakes. Five samples were collected from each quadrat and were mixed. Plant samples (n = 44) included whole plants for herbs, small shrubs, and leaves of large shrubs and trees. The plants in each soil quadrat were represented by the 3–5 common plants in the quadrat. Soil and plant samples were air-dried at a temporary location, transported in bags, freeze-dried, and ground in the laboratory before extraction.

### 2.2. Sample Extraction and Cleanup

In the laboratory, the soil and plant samples were transferred into polytetrafluoroethylene (PTFE) microwave tubes. 20 mL of hexane-acetone (1:1, *v*/*v*) mixture and surrogate standards (2-fluorobiphenyl and 4-terphenyl-d14) were added. The samples were soaked in the solution overnight until completely saturated. Then the PAHs were extracted using a microwave extraction system. The temperature was increased to 100 °C in 10 min, maintained for 10 min, and then cooled over 30 min. After filter pressing, 6 mL of a hexane-acetone (1:1, *v*/*v*) mixture was used to wash the extraction tube twice. The filtered extracts were rotary evaporated to approximately 1 mL. For solvent exchange, 5 mL of hexane was added, and the solution was rotary-evaporated to approximately 1 mL.

Anhydrous sodium-silica-alumina (1 cm, 10 cm, and 10 cm from top to bottom) chromatography columns were used for clean–up. Since plants contain chlorophyll, when purifying plant samples, 2 cm of Florisil was employed before alumina was used to enhance the purification effect. The concentrated solution was then transferred to a column. Then, 15 mL of hexane was added and the mixture was discharged. Subsequently, 70 mL of dichloromethane was added to elute the PAHs. The eluate containing the PAHs was concentrated to approximately 1 mL. Then, 5 mL of hexane were added for solvent exchange and the solution was rotary-evaporated to approximately 1 mL. Next, 25 μL internal standards (naphthalene-d8, acenaphthene-d10, chrysene-d12, perylene-d12 and anthracene-d10, 4 mg/L) was added. The solution was transferred into a 2 mL brown vial for instrument analysis.

### 2.3. Sample Analysis

An Agilent 6890 gas chromatograph (GC) coupled with a 5973 mass spectrometer detector (MSD) (Agilent Technologies Inc., Palo Alto, CA, USA) was used to determine the PAH contents of the samples. A DB-5 MS column (30 m*250 μm internal diameter, 0.25 μm film thickness) was used. The instruments were operated in electron impact (EI) and selective ion monitoring (SIM) modes. The carrier gas was helium (99.999%) and the flow rate was 1.2 mL/min. The injection volume was 1μL and the injection port temperature was 280 ℃. The temperature of ion source was 230 ℃. The oven temperature was programmed to an initial temperature of 60 ℃ for 1 min and then increased at a rate of 10 ℃/min from 60 to 180 ℃, at which point the temperature was maintained for 6 min. Then, the temperature increased to 250 ℃ at a rate of 10 ℃/min for 5 min and then increased to 305 ℃ at a rate of 15 ℃/min, and held for 6 min. The chromatograms of the 16 PAHs are shown in Figure S1.

Two parallel samples were used for soil and plant samples. The methods for recoveries were determined before the samples were formally analyzed. The recoveries of the 16 priority PAHs set by the US EPA are listed in Table S2. The recoveries of PAHs in soil were between 39.7% and 80.8%, and the recoveries of PAHs in plants ranged from 35.6 to 104%.

### 2.4. Data Analysis

Data processing and graphing were performed using Microsoft Excel 2016 (Microsoft, Seattle, WA, USA) and SPSS 20.0 (IBM, Armonk, NY, USA). Part of the graphing was performed using Origin 2020 (OriginLab, Northampton, MA, USA). Data normality was tested using the Shapiro-Wilk test, and data correlations were tested using the Spearman’s rank correlation coefficient and Pearson correlation coefficient.

## 3. Results

### 3.1. The Statistical Features of PAH Contents in Soil and Plants

Sixteen priority PAHs were detected in the soil and plant samples. A normality test of PAHs content was performed. The PAH contents in soil and plants were logarithmically transformed, and the Shapiro-Wilk test was used to test for a normal distribution in the data before and after transformation. In the soil samples, 12 PAHs did not follow a normal distribution (*p* < 0.05) before transformation, while 4 PAHs did follow a normal distribution (Nap, Flu, Phe and Ant). After logarithmic transformation, the skewness and kurtosis of the 16 PAHs were reduced, and all substances obeyed a lognormal distribution (Figure S2, Table S3). Therefore, the PAH monomer content of soil can be described by a lognormal distribution. Before transformation, only Ace showed a normal distribution in plant samples (*p* = 0.061 > 0.05). After logarithmic transformation, the skewness and kurtosis of the 16 PAHs were reduced. In addition to Ace, the other five PAHs (Nap, Phe, Ant, Chr and BaP) obeyed a normal distribution. However, in general, the contents of most PAH monomers did not obey a normal distribution before and after logarithmic transformation (Figure S3, Table S4). Therefore, it is not appropriate to describe the PAHs content of plants with normal or lognormal distributions.

In the soil samples, the average total PAH content was 190 ng/g, and the concentration range was 99.2–552 ng/g. The detection rates of the PAH monomers were all 100%, except for Ace and DahA, whose detection rates were 66.7% and 88.9%, respectively (Table 1). In plant samples, the average total content of PAHs was 275 ng/g, which was higher than that in soil. The total PAH content ranged from 123 ng/g to 743 ng/g. The detection rates of PAHs were 100%, except for six PAHs (Ace, BkF, BaP, IcdP, DahA, and BghiP). Phe was the most abundant PAH in soil and plants. In soil samples, the content of Any was the lowest, and the contents of Ace, DahA, and BaP were also low, and were even lower in plant samples (Tables S5 and S6).

### 3.2. Distribution and Composition of PAHs in Soil

**Figure 1:**
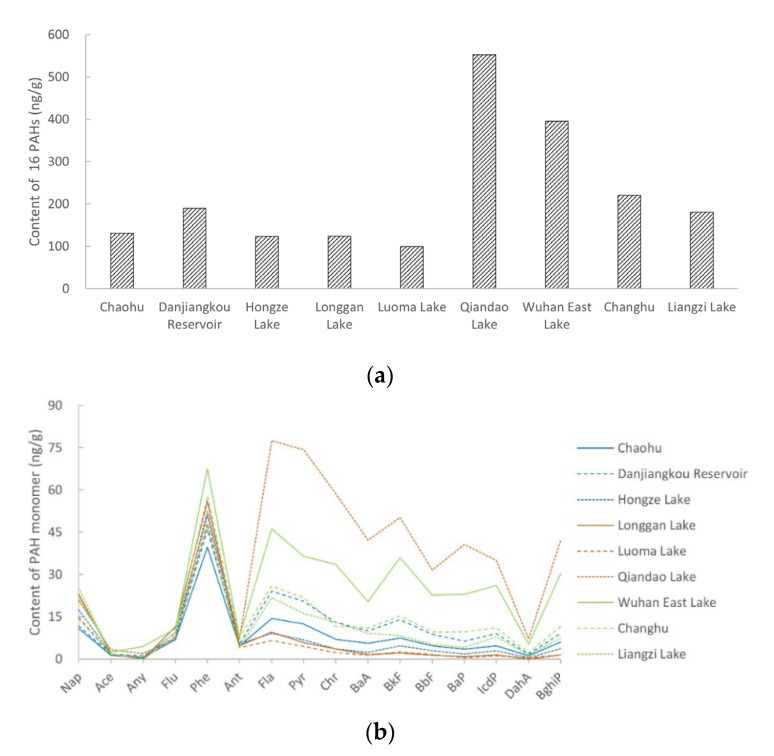
PAH contents in soil near the lake shores. (**a**) Total PAH content in soil; (**b**) PAH monomer content in soil.

The distribution and composition characteristics of PAHs in soils varied among different lakes. The PAH content of the soil sample from Qiandao Lake was the highest (552 ng/g), followed by the PAH content of the soil samples from Wuhan East Lake and Changhu. The PAHs contents of the soil samples from the other lakes were below 200 ng/g. The PAH content of the soil sample from Luoma Lake was the lowest, at only 99.2 ng/g (Figure 1a). The concentrations of Nap, Any, Ace, Flu, Phe, and Ant were similar among the different soil samples. The concentrations of other PAH monomers were higher in soil samples with higher total PAH contents (Figure 1b). The Spearman’s rank correlation coefficient was used to analyze the correlation between the PAH monomer content and total PAH content. The results showed no significant correlations between the Nap, Ace, Flu, Phe, and Ant contents and the total content of PAHs. However, the monomer contents of the other 11 PAHs and the total content were significantly correlated at a confidence level of 0.01, indicating that the differences in PAHs contents in soils from different lake areas were mainly due to the 11 PAHs.

**Figure 2:**
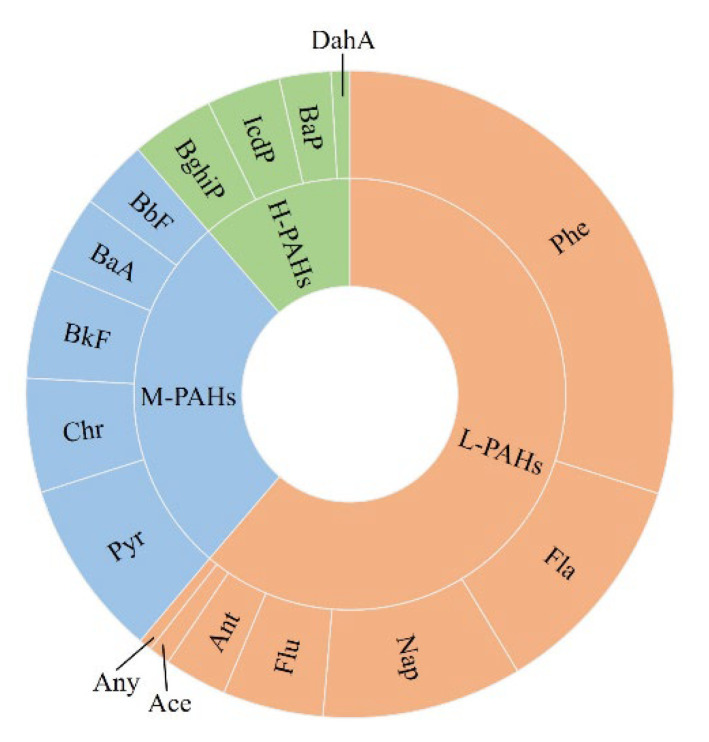
Ring chart of concentrations for 16 PAHs in soil near lake shores. L-PAHs: Low-molecular-weight PAHs. M-PAHs: Medium-molecular-weight PAHs. H-PAHs: High-molecular-weight PAHs.

Overall, Phe accounted for the highest proportion (30.0%) of the 16 PAHs in soil, followed by Flu, Nap, and Pyr, whereas BaP, DahA, Ace, and Any accounted for low proportions, all below 3% (Figure 2). The 16 PAHs were divided into three types: low-molecular-weight PAH (seven bi- and tri-ring PAHs), medium-molecular-weight PAH (five tetra-ring PAHs), and high-molecular-weight PAH (four penta- and hexa-ring PAHs). Low-molecular-weight PAHs accounted for 61.2% of the soil PAHs, while high-molecular-weight PAHs accounted for the smallest proportion of soil PAHs (11.4 %).

**Figure 3:**
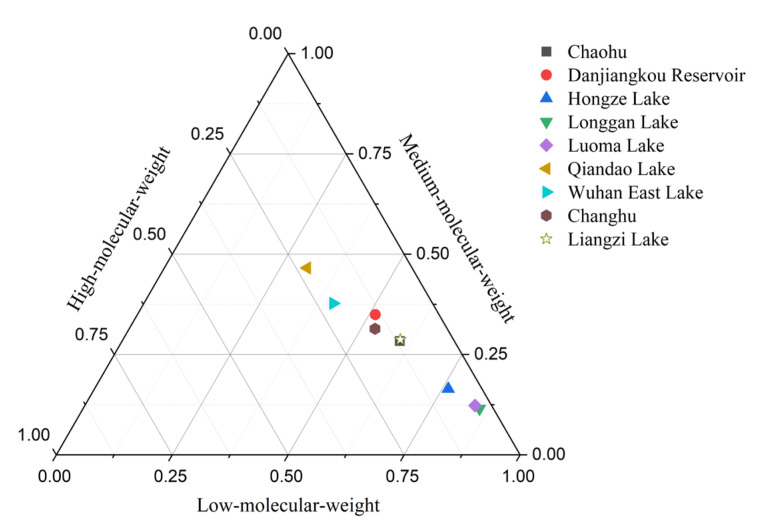
Composition of low-, medium- and high-molecular-weight PAHs in soil.

For soil samples from different lakes, the PAHs in the soil near Qiandao Lake were dominated by medium-molecular-weight PAHs, which accounted for 46.6% of the total PAHs, followed by low- and high-molecular-weight PAHs. Low-molecular-weight and medium-molecular-weight PAHs were the main PAHs in the soil of Wuhan East Lake. The proportions of the two groups were similar (40.8% and 37.7 %, respectively). The proportion of high-molecular-weight PAHs was the lowest. In the soils of the other seven lakes, low-molecular-weight PAHs were dominant, accounting for more than 50% of the total PAHs. In the soils of Hongze Lake, Longgan Lake, and Luoma Lake, low-molecular-weight PAHs accounted for more than 75% of the total PAHs. In general, the PAHs in the soils of the nine lakes were dominated by low- and medium-molecular-weight PAHs. High-molecular-weight PAHs accounted for the least number of PAHs (Figure 3).

### 3.3. Distribution and Composition of PAHs in Plants

**Figure 4:**
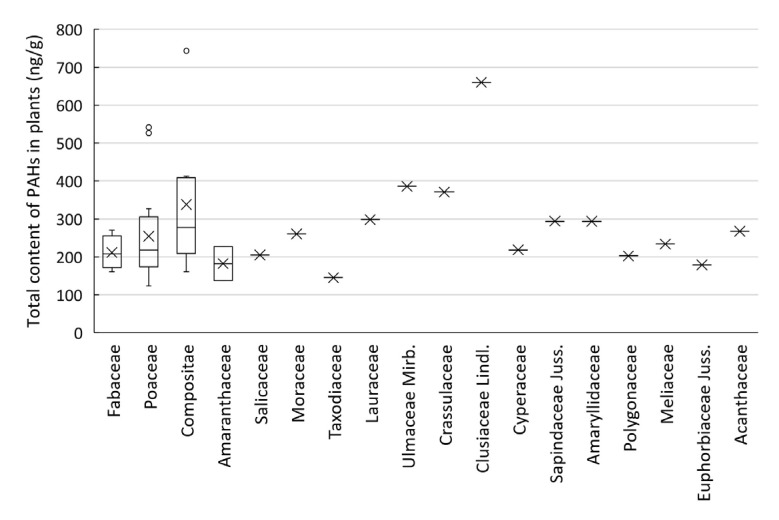
Total PAH contents in plants from different species. × means mean, ○ means outlier and - means median; except for Fabaceae, Poaceae, Compositae and Amaranthaceae, there was only one plant sample for other families.

There were also differences in the distributions and compositions of PAHs in different plants. The PAH content of the Garcinia genus Hypericum was the highest (660 ng/g), while the PAH content of plants from other families was less than 400 ng/g (Figure 4). Among the different species of plants, the highest PAH content was found in *Erigeron annuus* (L.) Pers. (743 ng/g). Two species of Poaceae also had two large values: *Setaria viridis* (L.) Beauv. (542 ng/g), and *Imperata cylindrica* (L.) Beauv. (527 ng/g). In general, except for a few extremely high values, the PAHs contents of different plant families ranged from 100 to 400 ng/g.

**Figure 5:**
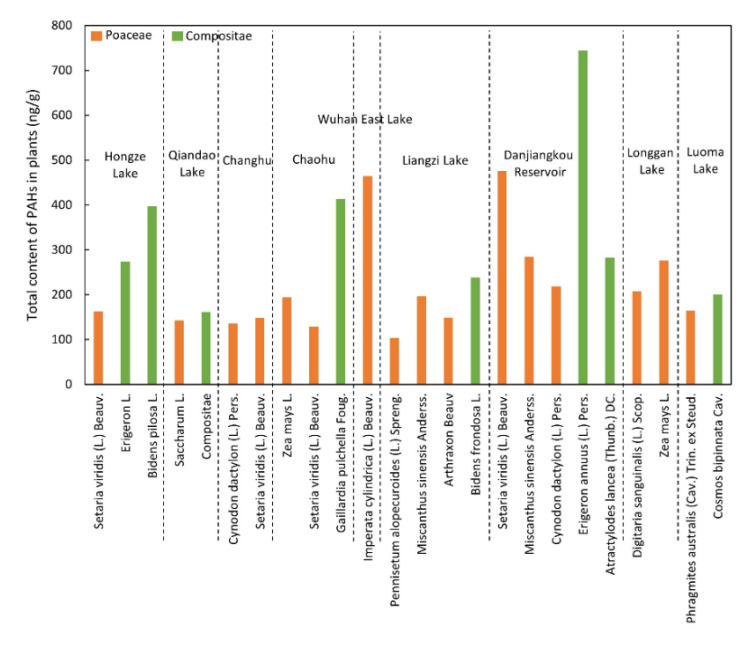
Total contents of PAHs in Poaceae (n = 16) and Compositae (n = 19) plants near 9 lakes.

Gramineae (n = 16) and Compositae (n = 8) plants with larger sample sizes were selected to compare the PAH contents in samples of the same family from different regions (Figure 5). The highest contents of PAHs in Gramineae and Compositae appeared in plants near the Danjiangkou Reservoir. Within Poaceae, the PAH content of *Imperata cylindrica* (L.) Beauv. near Wuhan East Lake was also high. The PAH contents of plants near lakes were all in the range of 100–200 ng/g and showed no obvious differences, except for plants from Wuhan East Lake, Danjiangkou Reservoir, and Longgan Lake. The difference in PAH content of Compositae from different regions was larger than that of Gramineae and ranged from 161 to 743 ng/g.

**Figure 6:**
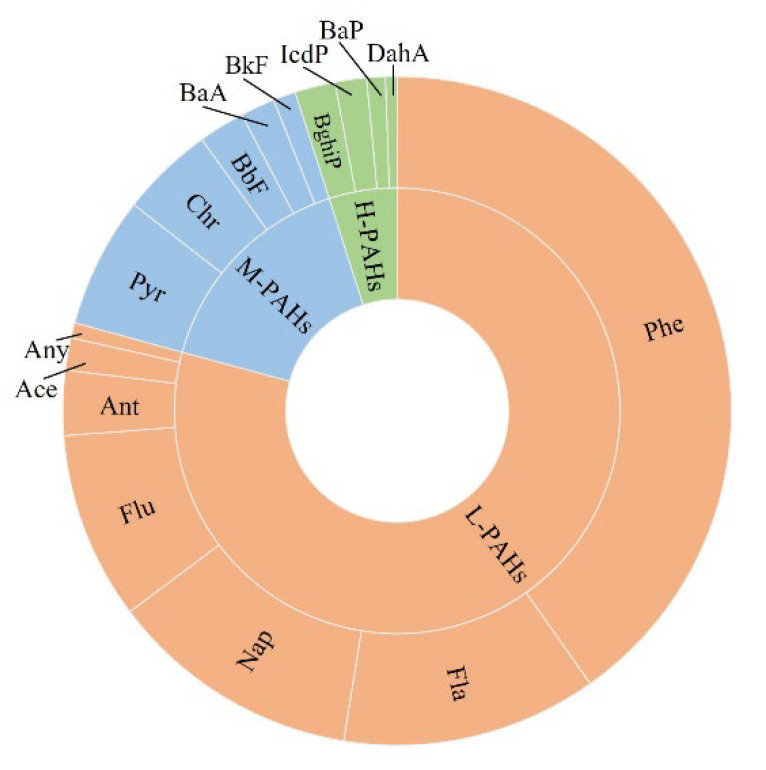
Ring chart of concentrations for 16 PAHs in plants near lake shores. L-PAHs: Low-molecular-weight PAHs. M-PAHs: Medium-molecular-weight PAHs. H-PAHs: High-molecular-weight PAHs.

The 16 PAHs in plants were dominated by Phe (40.2%), followed by Fla, Nap, and Flu, similar to the distribution of PAHs in the soil. DahA, Ace, and BaP accounted for relatively low proportions (all less than 1%) (Figure 6). Low-molecular-weight PAHs accounted for the highest proportion (79.2%), followed by medium-molecular-weight PAHs. High-molecular-weight PAHs accounted for the least proportion, which is consistent with composition of the soil.

**Figure 7:**
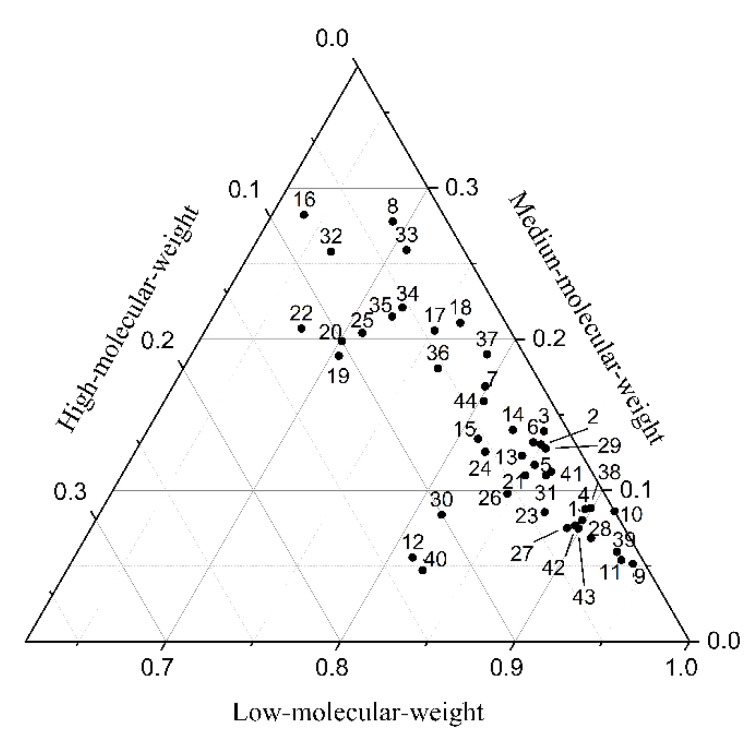
Compositions of PAHs in plants (See Table S6 in Supplementary Materials for plant numbers).

In different plant species, the compositions of PAHs were dominated by low-molecular-weight PAHs, the proportion of which was more than 60%. The proportion of high-molecular-weight PAHs was the lowest, all below 15%, and the proportion of medium-molecular-weight PAHs was below 30%. Except for the four plant samples, the proportion of medium-molecular-weight PAHs in the remaining plants was below 25% (Figure 7). In general, low-molecular-weight PAHs are dominant in different plants, followed by medium-molecular-weight PAHs. High-molecular-weight PAHs accounted for the least proportion. None of the high-molecular-weight PAHs in Compositae plants near Qiandao Lake (plant No. 10) reached the detection limit.

### 3.4. Possible sources of PAHs in soil and plants

PAHs are always emitted as mixtures, and different emission processes result in different compositions of PAHs. The diagnostic ratios of PAHs are widely used to identify potential sources of PAHs [30,31]. In this study, four diagnostic ratios, Ant/(Ant+Phe), BaA/(BaA+Chr), Fla/(Fla+Pyr) and IcdP/(IcdP+BghiP), were selected for source identification of PAHs. Typical values for the four diagnostic ratios are shown in Table 2.

**Figure 8:**
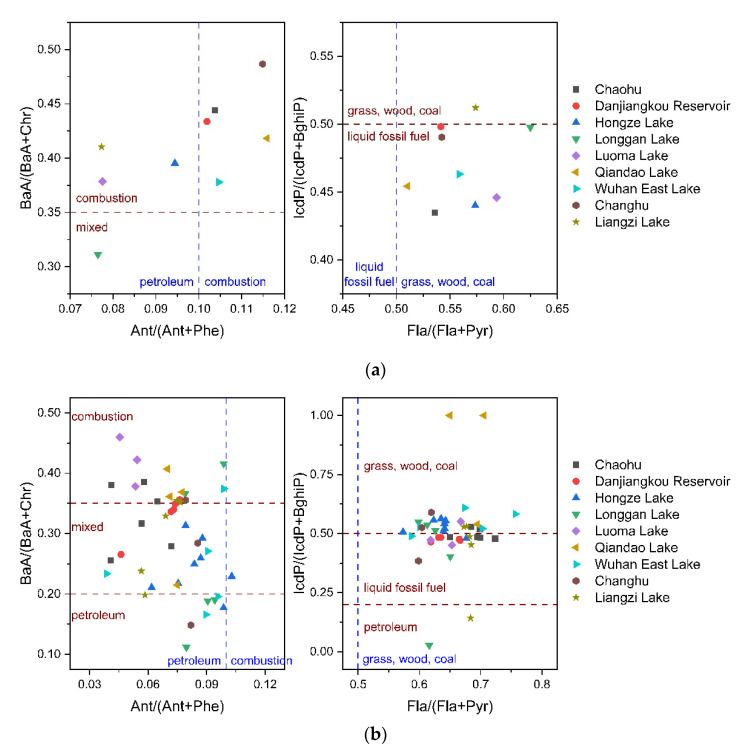
PAH diagnostic ratios of Ant/(Ant+Phe), BaA/(BaA+Chr), Fla/(Fla+Pyr) and IcdP/(IcdP+BghiP) in soil and plant samples. (**a**) Diagnostic ratios in soil. (**b**) Diagnostic ratios in plants.

The diagnostic ratios of PAHs in the samples are shown in Figure 8. 5 soil samples were found with the ratio of Ant/(Ant+Phe) > 0.1, suggesting the combustion source of PAHs, while the values of BaA/(BaA+Chr) showed that the PAHs in the soil samples were all from combustion sources except the PAHs in the soil sample near Longgan Lake. All soil samples were found with Fla/(Fla+Pyr) values > 0.5, indicating combustion sources of grass, wood and coal. However, the ratios of IcdP/(IcdP+BghiP) suggested that only the PAHs in the soil sample near Liangzi Lake came from the combustion of grass, wood and coal, while the PAHs in the other samples came from the combustion of liquid fossil fuels. In general, PAHs in soil samples were mainly from combustion sources.

For plant samples, almost all samples (97.7%) were found with Ant/(Ant+Phe) values < 0.1, suggesting petroleum sources, while the ratios of BaA/(BaA+Chr) showed that only 18.6% of samples were associated with petroleum sources. 100% of samples were found with Fla/(Fla+Pyr) > 0.5, 55.8% of samples were found with IcdP/(IcdP+BghiP) > 0.5, indicating the combustion of grass, wood and coal.

**Figure 9:**
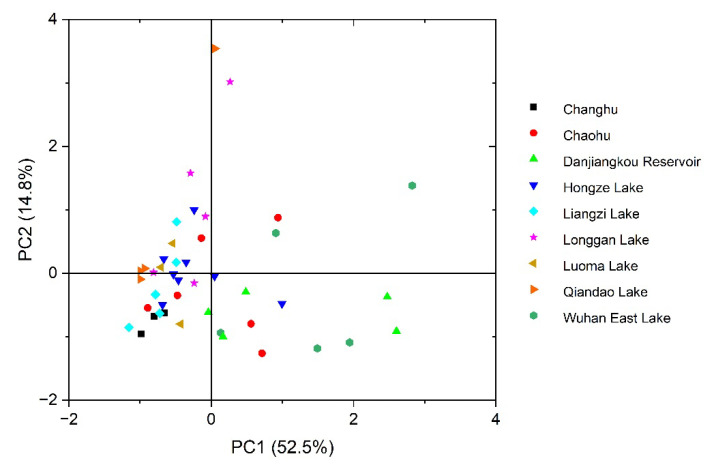
Scores of principal component 1 (PC 1) and principal component 2 (PC 2) for plants near different lakes.

To further investigate the possible sources of PAHs in plant samples, principal component analysis (PCA) was performed. Four principal components PC 1, PC 2, PC 3 and PC 4 with eigenvalue >1 were extracted and explained 52.5%, 14.8%, 9.62% and 7.19% (84.1% in total) of the total variance, respectively (Table 3). PC 1 was characterized by PAHs with four and five rings, indicating the contribution of coal and liquid fossil fuel. Pyr, Chr, BaA and BaP are markers of coal combustion [32,33]. BkF and BbF can be produced in diesel combustion [34]. PC 2 was loaded by Flu, Ace and DahA. Low-molecular-weight PAHs are the main PAHs emitted from coke ovens [35]. High-molecular-weight PAHs can be produced by lubricating oil combustion or industrial production [4]. Therefore, PC 2 represented the source of industrial processes. PC 1 and PC 2 together explained 67.3% of the total variance. Figure 9 shows the scores of PC1 and PC2 for all plant samples. PC 1 showed that the sources of PAHs in plants near Danjiangkou Reservoir and Wuhan East Lake were slightly different from those in plants near other lakes. However, in general, high similarity was found in scores for plant samples near different lakes, indicating similarity in the sources of PAHs.

### 3.5. Relationship between the Distribution and Composition of PAHs in Soil and Plants

#### 3.5.1. Correlation between the distribution of PAHs in soil and plants

The PAH contents in 44 plant samples and the soil in which they grew were obtained. At a single location, the PAH monomers that were higher in the soil were generally higher in the plants (Figure S4). The Spearman’s rank correlation coefficient test showed that there was a significant correlation between the PAH monomer content of most plants and that of soil at the same location (Figure S5, Table S7). Therefore, the distribution of different PAH monomers may have similar trends in both soil and plants.

The correlation between the PAH contents of soil and plants was analyzed using the Spearman′s rank correlation coefficient. The results showed that there was no significant correlation between the total content of PAHs in soil and plants, and the same was true for the contents of high- medium-, and low-molecular-weight PAHs. Among the 16 PAH monomers, only Ace, Ant, BkF, BbF, and DahA showed significant correlations between the soil and plant samples (Table 4). The reason for the poor correlations may be the influence of plant species. Different plant species may have different PAH absorption and transformation processes. Plants also synthesize small amounts of endogenous PAHs, which may cause insignificant results.

#### 3.5.2. Accumulation of PAHs in Plants

The plant concentration factor (PCF) was calculated by dividing the concentration of PAHs in plants by the concentration of PAHs in soil to characterize the accumulation of PAHs in plants. According to the PCF values, two species of Compositae, *Bidens pilosa* L. and *Gaillardia pulchella* Foug., showed relatively strong accumulation ability of Chr and Any, respectively. The *Zea mays* L. (corn) near Longgan Lake was found with a relatively higher PCF value of BghiP, but the corn near Chaohu did not show this characteristic. The PCF values of the four plants are shown in Table 5.

#### 3.5.3. Effects of Physicochemical Properties on the Distribution of PAHs in Soil and Plants

**Figure 10:**
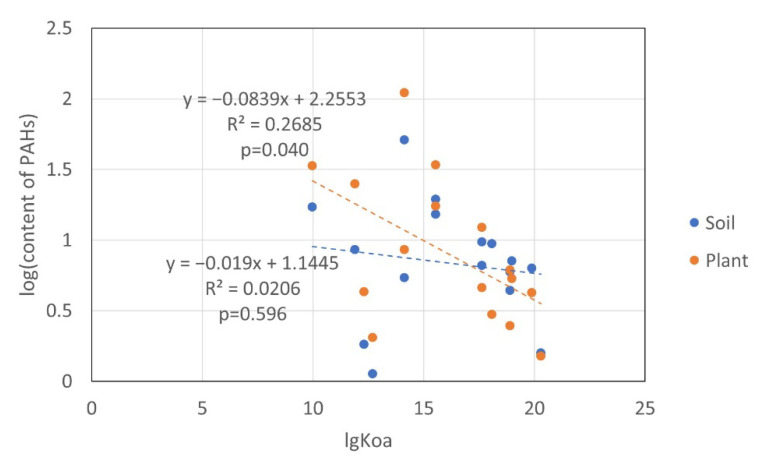
Relationship between the PAH contents and Koa.

The physical and chemical properties of PAHs affect their environmental distribution. In this study, correlations between the PAH monomer contents in soil and plants and their octanol-water partition coefficients (Kow) and octanol-gas partition coefficients (Koa) were analyzed. The Pearson’s correlation coefficient was used to measure the correlation. There was no significant correlation between the PAH monomer content (logarithmic value) and lgKow values in either soil or plants. The PAH monomer content of plants (logarithmic value) had a significant negative correlation with lgKoa; that is, high-molecular-weight PAHs with higher lgKoa values were less abundant in plants. However, there was no significant correlation between the PAH monomer content (logarithmic value) and lgKoa values in soil (Table 6). The linear fit between the PAH monomer content in plants (logarithmic value) and lgKoa was stronger than that of soil (R^2^_plant_ = 0.2685 > R^2^_soil_ = 0.0206), indicating that the correlation between Koa and the PAH content in plants was stronger (Figure 10). Plants contain lipids, which may lead to a significant correlation between the content of PAHs in plants and Koa, whereas the content of PAHs in soil had no significant correlation with Koa. There is no significant correlation between the PAH monomer content in plants and their Kow, possibly because the mechanism by which plants absorb PAHs from soil was complex, not just distribution.

## 4. Discussion

The contents of 16 PAHs in the samples were determined. The highest concentrations of Phe were found in soil and plants. On the one hand, Phe is the second most emitted PAH in China, after Nap [36]. On the other hand, medium to low volatility atmospheric PAHs are more prone to wet and dry deposition [37], so Phe with lower volatility is more likely to enter soil and plants than Nap. For PAHs in soil, Phe is also dominant in rural soils in China [38] and in soils near Qinghai Lake [39]. In urban soils in China [31], soils of the Yangtze River Delta [40] and soils of the Yinma River Basin [41], the content of Phe is not the highest, but it still ranks in the forefront. For PAHs in plants, the dorminance of Phe has also been found in other studies [42,43].

The PAHs content in the soil near Qiandao Lake was the highest among the 9 soil samples, which may be caused by the proximity of Qiandao Lake to the main traffic road. The diagnostic ratio BaA/(BaA+Chr) of the soil sample near Qiandao Lake was above 0.35, indicating the source of vehicular emission of PAHs [44]. Liyang-Ningde Expressway and Shanghai-Jiaxing-Huzhou Interprovincial Expressway both pass near Qiandao Lake, and vehicle exhaust emissions may lead to the accumulation of PAHs [45]. Higher traffic volume can cause higher PAH contents in roadside soils [46]. The PAH contents in soil decrease with increasing distance from the road, but significant concentrations still can be found [47].

Sources of PAHs in the samples were identified using diagnostic ratios. The results of the four diagnostic ratios were inconsistent, which was also reported in other studies [31,48,49]. It may be due to the different degradation rates of PAHs during transport processes [50]. The air concentrations and molecular ratios of PAHs will change as the distance from the source increases [51]. Oxidation and biodegradation processes in the soil can also lead to changes in diagnostic rates [52]. Furthermore, considering that the accumulation of PAHs in plants can lead to changes in diagnostic ratios, caution is required when using diagnostic ratios in plants [53,54]. For these reasons, some authors have criticized the unquestioning application of PAH diagnosis ratios [55,56]. To further investigate the source of PAHs, PCA analysis was performed on plant samples. Figure 9 shows that spatial location had little effect on sources of PAHs in plants, indicating the effect of plant species on PAHs accumulation. However, PCA analysis was not available for PAHs in soil in our study due to the small amount of soil samples (n < 16). Compound-specific isotope analysis is another method for PAH source identification [57,58]. It can avoid the adverse effects of the selective degradation of PAHs, but suffers from overlapping of end-members for several sources [59]. The combined application of different methods will provide more valuable information on the sources of PAHs.

Phytoextraction is one of the main approaches for the removal of organic compounds from contaminated soils [60]. BCF values indicated the potential of *Bidens pilosa* L. and *Gaillardia pulchella* Foug in the Compositae family and cron in the Poaceae family for phytoremediation. The corn near Longgan Lake showed a relatively strong accumulation of BghiP, which is consistent with the study on corn in Changchun, China [61]. However, this accumulation characteristic was not reflected in corn near Chaohu, nor in corn grown in soil treated with sewage sludge [62]. The presence of other plants at the site may affect the accumulation of PAHs in the target plants [63]. Soil characteristics and the presence of other contaminants can also affect the uptake [64,65]. In addition to absorbing PAHs from the soil, corn can also absorb PAHs from the atmosphere through leaves [66], which may also contribute to differences in PCF values at different locations. More research is needed to determine the practicability of the three plants for phytoremediation. The tolerance of plants to PAHs pollution also needs to be considered when applied to remediation.

## 5. Conclusions

Plants can absorb PAHs from various aspects of the environment, such as the atmosphere, water, and soil. In this study, GC-MS was used to determine the contents of 16 priority PAHs in soil and typical plants near nine major lakes in eastern China to explore the distribution and correlation of PAHs in soil and plants.

The average total PAH content of the soil samples was 190.35 ng/g, and the concentration range was 99.17–552.10 ng/g. The content of PAHs in plants was higher than that in soil, with an average total content of 274.66 ng/g and a concentration range of 122.93–743.44 ng/g. In terms of composition, the PAHs in soil were dominated by medium- and low-molecular-weight PAHs, whereas the PAHs in plants were dominated by low-molecular-weight PAHs. Diagnostic ratios and PCA identified combustion source as the main source of PAHs in soil and plants.

The PAH monomer content of plants was negatively correlated with their physicochemical properties, Koa. Correlation analysis indicated that the correlation between PAH contents in plants and soil was weak. This may be caused by the selective accumulation of plants. PCF values showed *Bidens pilosa* L. and *Gaillardia pulchella* Foug in the Compositae family and cron in the Poaceae family with a relatively stronger accumulation of PAHs, indicating their potential for phytoremediation. Our work complements studies on PAH pollution in soil and plants and presents new possibilities for species used for phytoremediation.

## Figures and Tables

**Table 1:** Statistical features of PAH contents in soil and plants near the lake shores (ng/g).

PAHs ^2^	Mean		Median		Maximum		Minimum		Detection Rate	
	Soil	Plant	Soil	Plant	Soil	Plant	Soil	Plant	Soil	Plant
Nap	17.1	33.5	17.5	31.2	25.3	61.4	10.9	17.2	100%	100%
Ace	1.83	4.29	1.71	4.13	3.25	6.59	1.30	2.65	100%	100%
Any	1.13	2.04	0.50	1.27	4.46	14.7	ND ^1^	ND ^1^	66.7%	88.6%
Flu	8.57	24.9	8.11	22.6	11.5	47.8	6.86	14.3	100%	100%
Phe	51.2	110	50.9	105	67.6	226	39.7	57.1	100%	100%
Ant	5.39	8.57	5.22	8.12	7.90	20.8	3.96	4.06	100%	100%
Fla	19.5	34.0	21.8	19.4	77.5	208	6.56	7.39	100%	100%
Pyr	15.2	17.4	16.2	10.5	74.3	104	4.49	3.55	100%	100%
Chr	9.72	12.3	11.6	8.79	58.7	49.2	2.27	1.52	100%	100%
BaA	6.63	4.61	9.10	2.69	42.2	18.2	1.38	0.98	100%	100%
BkF	9.42	2.98	8.33	2.14	50.2	10.2	2.11	ND ^1^	100%	93.2%
BbF	5.94	6.14	5.25	3.69	31.6	20.9	1.33	0.95	100%	100%
BaP	4.39	2.48	4.11	1.36	40.6	11.7	0.52	ND ^1^	100%	81.8%
IcdP	6.33	4.25	7.75	2.45	35.0	20.0	1.19	ND ^1^	100%	97.7%
DahA	1.59	1.50	1.42	ND ^1^	7.15	34.6	ND ^1^	ND ^1^	88.9%	31.8%
BghiP	7.12	5.32	7.38	2.46	42.0	41.2	1.46	ND ^1^	100%	93.2%
$\sum_{16}\mathrm{PAHs}$ ^3^	190	275	181	234	552	743	99.2	123		

^1^ ND means not detected. ^2^ Nap—Naphthalene, Ace—Acenaphthene, Any—Acenaphthylene, Flu—Fluorene, Phe—Phenanthrene, Ant—Anthracene, Fla—Fluoranthene, Pyr—Pyrene, Chr—Chrysene, BaA—Benz[a]anthracene, BkF—Benzo[k]fluoranthene, BbF—Benzo[b]fluoranthene, BaP—Benzo[a]pyrene, IcdP—Indeno[1,2,3-cd]pyrene, DahA—Dibenzo[a,h]anthracene, BghiP—Benzo[g,h,i]perylene. ^3^ $\sum_{16}\mathrm{PAHs}$ means the sum of 16 PAHs.

**Table 2:** Typical values for Ant/(Ant+Phe), BaA/(BaA+Chr), Fla/(Fla+Pyr) and IcdP/(IcdP+BghiP).

Source	Ant/(Ant+Phe)	BaA/(BaA+Chr)	Fla/(Fla+Pyr)	IcdP/(IcdP+BghiP)
Petroleum	<0.1	<0.2	<0.4	<0.2
Combustion	>0.1	>0.35		
Liquid fossil fuel combustion			0.4–0.5	0.2–0.5
Grass, wood, coal combustion			>0.5	>0.5

**Table 3:** Component matrix for principal component analysis (PCA) of PAHs in plant samples.

	Principal Component			
	1	2	3	4
Nap	0.46	0.43	−0.30	−0.36
Ace	0.61	0.61	−0.053	−0.27
Any	0.46	0.17	0.63	0.39
Flu	0.52	0.77	0.18	0.15
Phe	0.84	0.24	0.30	−0.16
Ant	0.65	0.46	−0.40	−0.13
Fla	0.77	−0.25	0.43	−0.31
Pyr	0.84	−0.22	0.29	−0.35
Chr	0.84	−0.34	0.076	−0.20
BaA	0.92	−0.29	0.008	−0.044
BkF	0.89	−0.24	−0.19	0.22
BbF	0.92	−0.31	−0.12	0.11
BaP	0.86	−0.21	−0.21	0.35
IcdP	0.84	−0.20	−0.27	0.37
DahA	0.15	0.53	0.35	0.37
BghiP	0.50	0.29	−0.44	0.12

**Table 4:** Correlation coefficient between PAH monomer content in soil and plants.

		In Soil															
		Nap	Ace	Any	Flu	Phe	Ant	Fla	Pyr	Chr	BaA	BkF	BbF	BaP	IcdP	DahA	BghiP
**In** **palnts**	Nap	−0.042	0.057	0.180	0.223	0.275	0.279	0.028	−0.037	−0.035	−0.037	−0.117	−0.117	−0.037	−0.037	−0.034	−0.034
	Ace	0.201	0.277	0.109	0.255	0.158	0.135	0.013	−0.028	0.057	−0.028	−0.054	−0.054	−0.028	−0.028	−0.048	−0.048
	Any	−0.151	−0.008	0.383 *	−0.137	−0.042	0.106	0.297	0.285	0.317 *	0.285	0.109	0.109	0.285	0.285	0.303 *	0.303 *
	Flu	0.149	0.129	0.009	0.240	0.063	0.027	−0.101	−0.135	−0.065	−0.135	−0.052	−0.052	−0.135	−0.135	−0.170	−0.170
	Phe	−0.013	0.025	0.097	0.122	−0.076	0.015	−0.063	−0.072	0.007	−0.072	−0.060	−0.060	−0.072	−0.072	−0.095	−0.095
	Ant	0.216	0.257	0.124	0.553 **	0.327 *	0.370 *	−0.068	−0.077	−0.035	−0.077	−0.458 **	−0.458 **	−0.077	−0.077	−0.140	−0.140
	Fla	−0.313 *	−0.110	0.343 *	−0.052	−0.235	0.101	0.035	0.086	0.170	0.086	−0.185	−0.185	0.086	0.086	0.092	0.092
	Pyr	−0.280	−0.128	0.293	0.082	−0.134	0.148	−0.025	0.015	0.080	0.015	−0.299 *	−0.299 *	0.015	0.015	0.017	0.017
	Chr	−0.205	−0.066	0.399 **	0.140	0.014	0.284	0.152	0.185	0.212	0.185	−0.394 **	−0.394 **	0.185	0.185	0.171	0.171
	BaA	−0.388 **	−0.207	0.417 **	−0.031	−0.148	0.199	0.114	0.174	0.201	0.174	−0.299 *	−0.299 *	0.174	0.174	0.187	0.187
	BkF	−0.327 *	−0.179	0.427 **	0.119	0.003	0.289	0.091	0.129	0.139	0.129	−0.391 **	−0.391 **	0.129	0.129	0.132	0.132
	BbF	−0.386 **	−0.244	0.423 **	0.050	−0.091	0.234	0.089	0.128	0.146	0.128	−0.345 *	−0.345 *	0.128	0.128	0.129	0.129
	BaP	−0.491 **	−0.301 *	0.485 **	−0.152	−0.194	0.161	0.168	0.195	0.219	0.195	−0.145	−0.145	0.195	0.195	0.216	0.216
	IcdP	−0.416 **	−0.277	0.441 **	−0.137	−0.215	0.106	0.190	0.235	0.259	0.235	−0.182	−0.182	0.235	0.235	0.255	0.255
	DahA	−0.383 *	−0.098	0.510 *	−0.392 *	−0.255	0.127	0.345 *	0.358 *	0.403 **	0.358 *	0.186	0.186	0.358 *	0.358 *	0.358 *	0.358 *
	BghiP	−0.261	−0.278	0.239	−0.064	−0.217	−0.067	0.114	0.100	0.144	0.100	−0.059	−0.059	0.100	0.100	0.109	0.109

* indicates that the correlation is significant at a confidence level of 0.05; ** indicates that the correlation is significant at a confidence level of 0.01.

**Table 5:** Values of plant concentration factor (PCF) of PAHs in *Zea mays* L. (corn), *Bidens pilosa* L. and *Gaillardia pulchella* Foug.

	Plant Concentration Factor (PCF)			
	*Bidens pilosa* L.	*Gaillardia pulchella* Foug.	Corn near Longgan Lake	Corn near Chaohu
Nap	1.97	2.35	1.99	2.30
Ace	3.38	2.85	3.32	2.89
Any	3.23	**29.2**	− ^1^	6.11
Flu	2.75	6.96	3.93	2.98
Phe	2.79	4.66	2.55	2.67
Ant	1.77	1.73	3.37	1.38
Fla	6.01	3.73	1.39	1.69
Pyr	6.02	1.85	1.45	0.855
Chr	**11.7**	1.49	0.586	0.741
BaA	4.77	1.15	0.923	0.430
BkF	1.08	0.570	0.511	0.273
BbF	3.94	1.67	1.87	0.902
BaP	1.73	1.57	0.00	0.528
IcdP	1.60	1.46	0.805	0.719
DahA	0.00	2.50	0.00	0.00
BghiP	1.22	1.20	**28.3**	0.588

^1^ Any was not detected in soil near Longgan Lake.

**Table 6:** Correlation between the contents of PAHs in soil and plants and their Kow and Koa values.

		lg(PAHs in Soil)	lg(PAHs in Plants)
lgKow	r	−0.071	−0.461
	*p*	0.794	0.073
lgKoa	r	−0.143	−0.518 *
	*p*	0.596	0.040

* Indicates that the correlation is significant at a confidence level of 0.05.

## Data Availability

Not applicable.

## Supplementary Materials

The following supporting information can be downloaded at: https://www.mdpi.com/article/10.3390/toxics10100577/s1, Table S1: Detailed information about the 9 major lakes from eastern China; Figure S1: Chromatograms of a standard solution (1000ng/mL) of 16 PAHs; Table S2: Recoveries of 16 PAHs in soil and plants; Figure S2: Frequency distribution histogram of PAHs in soil before (a) and after (b) logarithmic transformation; Table S3: Skewness, kurtosis and normal distribution test results of PAHs in soil before and after logarithmic transformation; Figure S3: Frequency distribution histogram of PAHs in plants before (a) and after (b) logarithmic transformation; Table S4: Skewness, kurtosis and normal distribution test results of PAHs in plants before and after logarithmic transformation; Table S5: Contents of PAHs in soil near each lake (ng/g); Table S6: Contents of PAHs in plants near each lake (ng/g); Figure S4: Contents of PAH monomers in soil and plants near different lakes; Figure S5: Correlation of PAH contents in soil and plants near different lakes; Table S7: Correlation coefficient, p, linear fitting function and R2 of PAH monomer contents in soil and plants in different lake areas.
