# Supplementary material for: Distribution and Relationships of Polycyclic Aromatic Hydrocarbons (PAHs) in Soils and Plants near Major Lakes in Eastern China
Source: Toxics. 2022 Sep 30;10(10):577. doi: 10.3390/toxics10100577 (PMC9607041; doi:10.3390/toxics10100577)
Supplement: Supplementary file 1 [file toxics-10-00577-s001.zip › toxics-1894720-supplementary.pdf]

Distribution and relationships of polycyclic aromatic hydrocarbons (PAHs) in soils and plants near major lakes in eastern China

Contents:

Table S1. Detailed information about the 9 major lakes from eastern China.

Figure S1. Chromatograms of a standard solution (1000ng/mL) of 16 PAHs.

Table S2. Recoveries of 16 PAHs in soil and plants.

Figure S2. Frequency distribution histogram of PAHs in soil before (a) and after (b) logarithmic transformation.

Table S3. Skewness, kurtosis and normal distribution test results of PAHs in soil before and after logarithmic transformation.

Figure S3. Frequency distribution histogram of PAHs in plants before (a) and after (b) logarithmic transformation.

Table S4. Skewness, kurtosis and normal distribution test results of PAHs in plants before and after logarithmic transformation.

Table S5. Contents of PAHs in soil near each lake (ng/g).

Table S6. Contents of PAHs in plants near each lake (ng/g).

Figure S4. Contents of PAH monomers in soil and plants near different lakes.

Figure S5. Correlation of PAH contents in soil and plants near different lakes.

Table S7. Correlation coefficient, p, linear fitting function and R2 of PAH monomer contents in soil and plants in different lake areas.

Table S1. Detailed information about the 9 major lakes from eastern China.

Lake	Province	Basin	Precipitation	Climatic zone
Chaohu	Anhui	Middle and lower reaches of the Yangtze River	Humid area	Northern subtropic zone
Changhu	Hubei			
Danjiangkou Reservoir	Hubei			
Wuhan East Lake	Hubei			
Longgan Lake	Hubei			
Liangzi Lake	Hubei			
Qiandao Lake	Zhejiang			
Hongze Lake	Jiangsu	Huaihe River Basin	Semi-humid area	Warm temperate zone
Luoma Lake	Jiangsu	Huaihe River Basin	Semi-humid area	Warm temperate zone

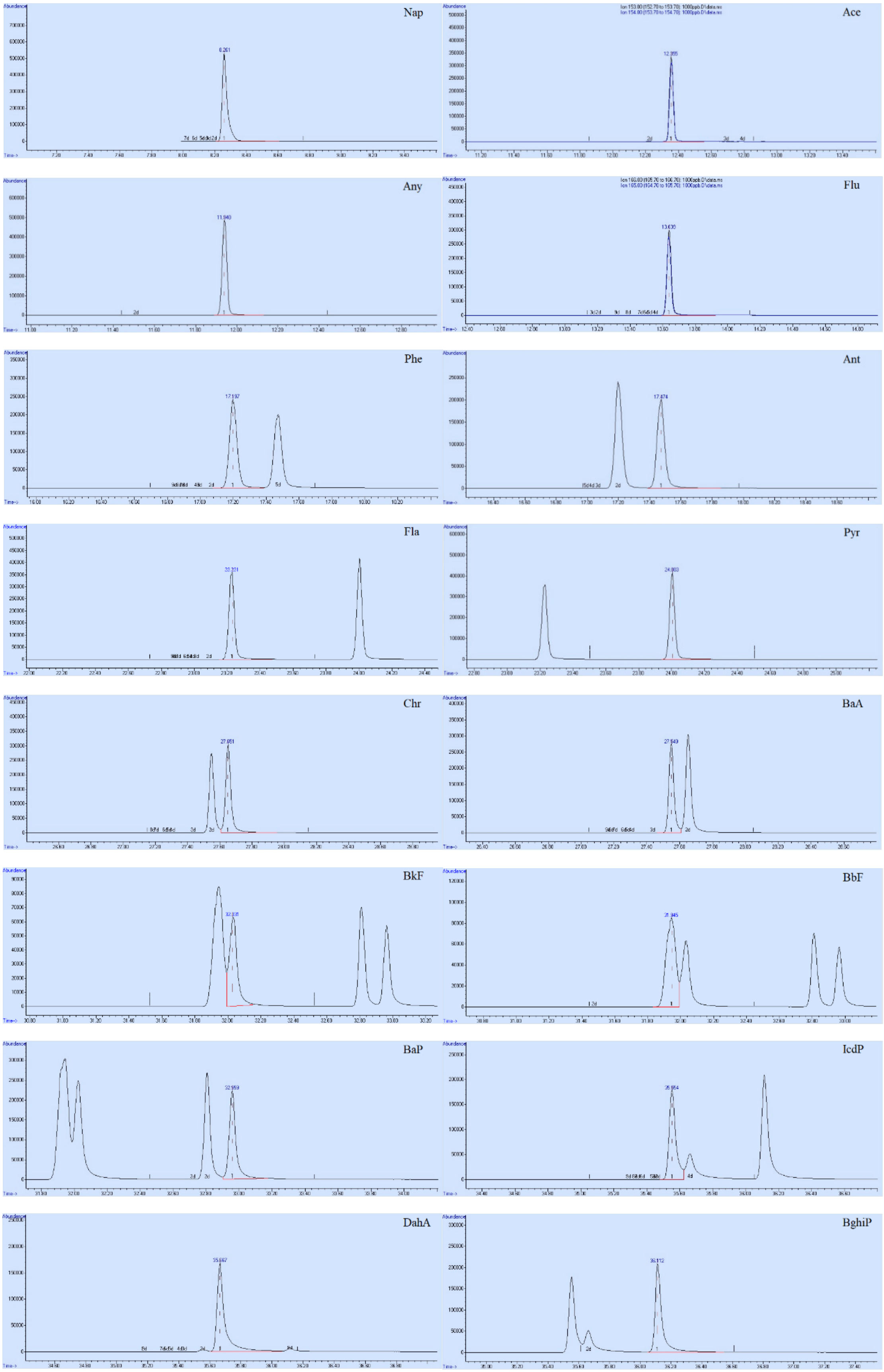

Figure S1. Chromatograms of a standard solution (1000ng/mL) of 16 PAHs.

Table S2. Recoveries of 16 PAHs in soil and plants.

PAHs	Short name	Recovery in soil/%	Recovery in plants/%
Naphthalene	Nap	39.7	71.9
Acenaphthene	Ace	65.0	84.5
Acenaphthylene	Any	61.0	84.9
Fluorene	Flu	70.7	91.8
Phenanthrene	Phe	67.8	103.7
Anthracene	Ant	71.5	88.4
Fluoranthene	Fla	74.2	90.3
Pyrene	Pyr	80.8	99.4
Chrysene	Chr	80.1	104.1
Benz[a]anthracene	BaA	77.2	101.3
Benzo[k]fluoranthene	BkF	73.8	65.9
Benzo[b]fluoranthene	BbF	46.5	35.6
Benzo[a]pyrene	BaP	57.7	76.2
Indeno[1,2,3-cd]pyrene	IcdP	54.4	85.3
Dibenzo[a,h]anthracene	DahA	56.4	79.1
Benzo[g,h,i]perylene	BghiP	59.1	70.6

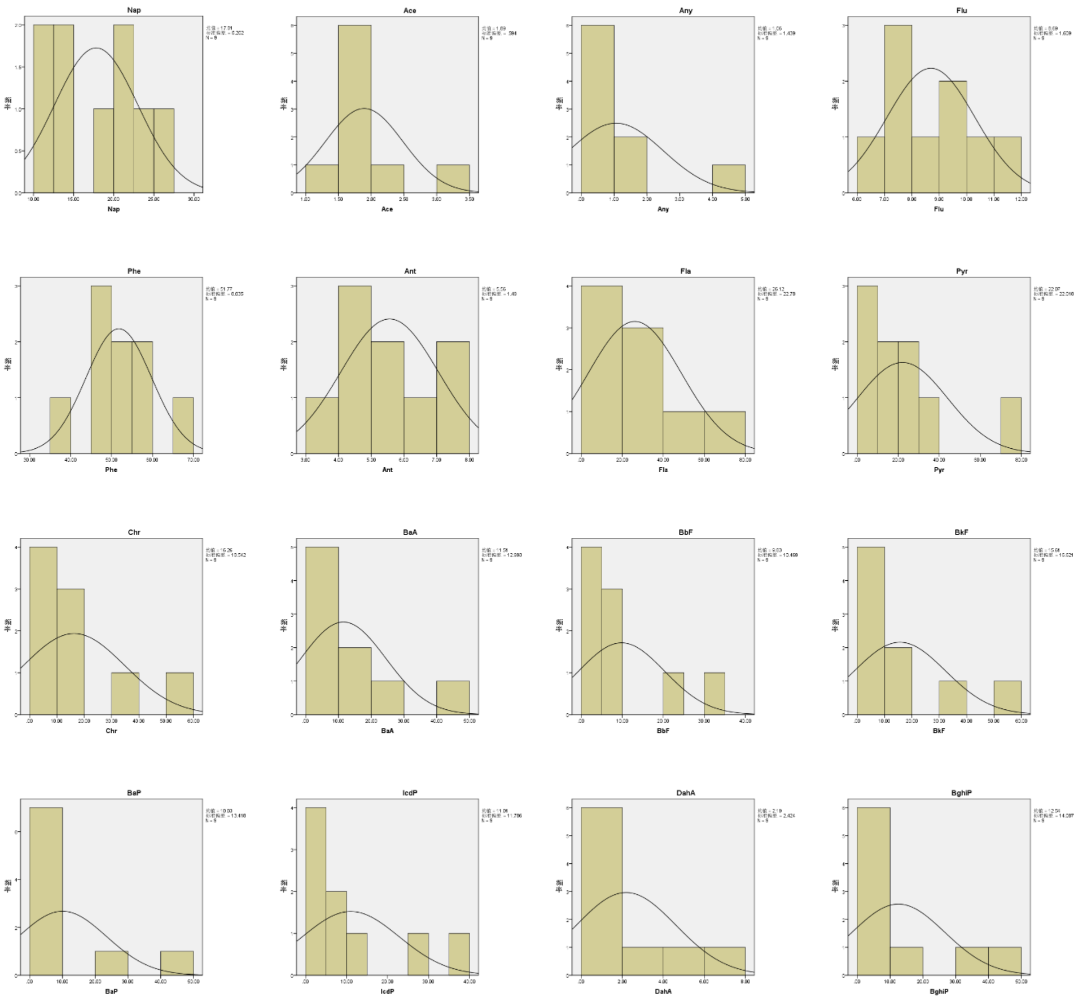
Nap
Ace
Any
Flu
Phe
Ant
Fla
Pyr
Chr
BaA
BbF
BkF
BaP
IcdP
DahA
BghiP

before (a)

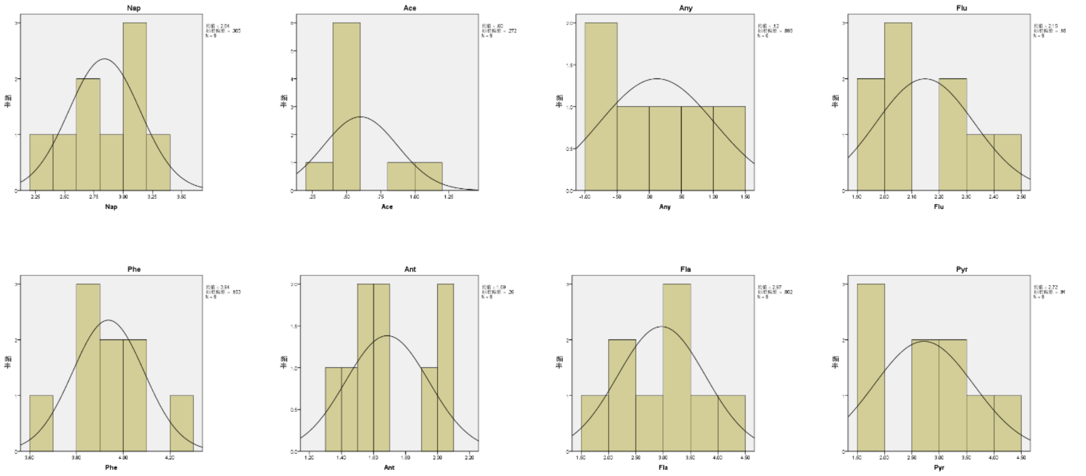
Nap
Ace
Any
Flu
Phe
Ant
Fla
Pyr

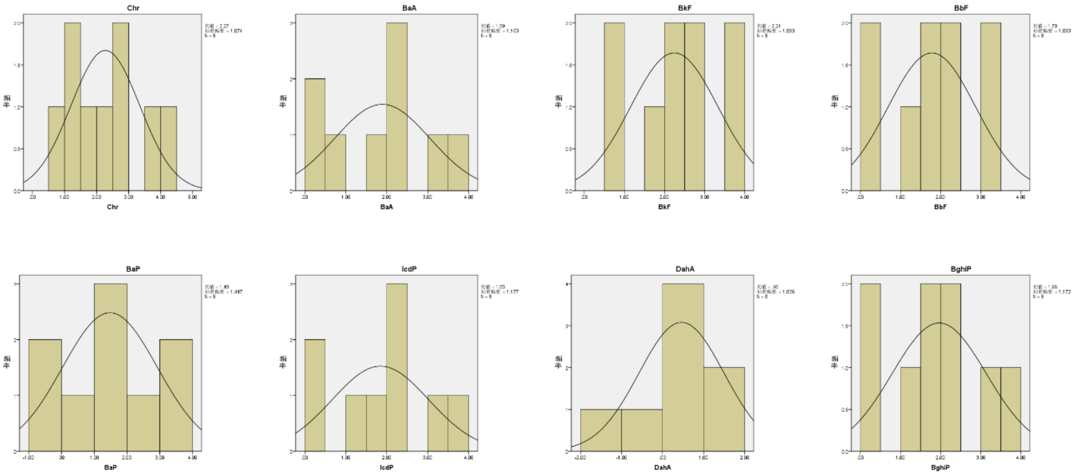

after (b)

Figure S2. Frequency distribution histogram of PAHs in soil before (a) and after (b) logarithmic transformation.

Table S3. Skewness, kurtosis and normal distribution test results of PAHs in soil before and after logarithmic transformation.

PAHs	Before			After(ln)		
	Skewness	Kurtosis	p	Skewness	Kurtosis	p
Nap	9.56	0.04	**0.565**	-0.256	-1.479	0.516
Ace	1.818	3.266	0.011	1.344	1.884	0.087
Any	1.941	4.051	0.009	0.429	-0.426	0.875
Flu	0.687	-0.774	**0.366**	0.479	-1.078	0.533
Phe	0.670	1.037	**0.876**	0.230	0.667	0.988
Ant	0.639	-1.295	**0.152**	0.422	-1.436	0.271
Fla	1.704	2.860	0.026	0.379	-0.557	0.822
Pyr	1.976	4.243	0.010	0.323	-0.476	0.852
Chr	1.865	3.229	0.005	0.339	-0.673	0.691
BaA	1.953	4.117	0.025	0.014	-0.945	0.663
BkF	1.504	1.370	0.018	0.146	-0.904	0.779
BbF	1.505	1.371	0.018	0.145	-0.903	0.779
BaP	1.870	3.103	0.004	0.036	-0.758	0.949
IcdP	1.431	1.074	0.024	-0.036	-0.989	0.776
DahA	1.448	1.163	0.028	0.016	-0.587	0.844
BghiP	1.570	1.503	0.010	0.055	-0.726	0.637

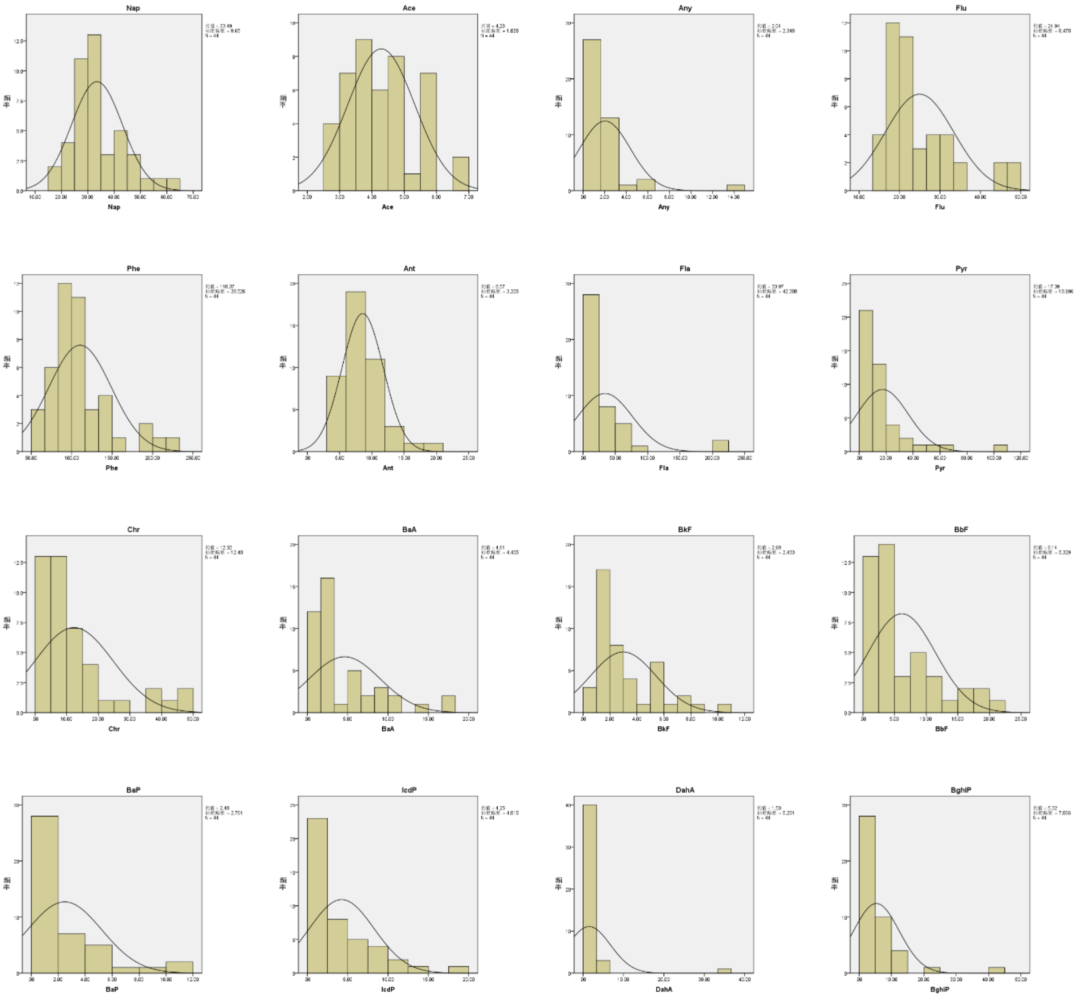
Nap
Ace
Any
Flu
Phe
Ant
Fla
Pyr
Chr
BaA
BkF
BbF
BaP
IcdP
DahA
BghiP

before (a)

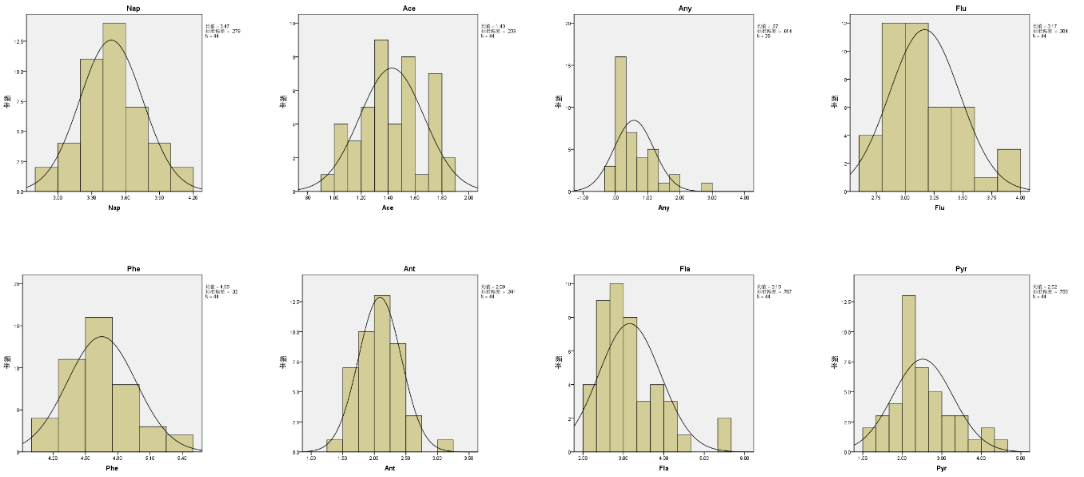
Nap
Ace
Any
Flu
Phe
Ant
Fla
Pyr

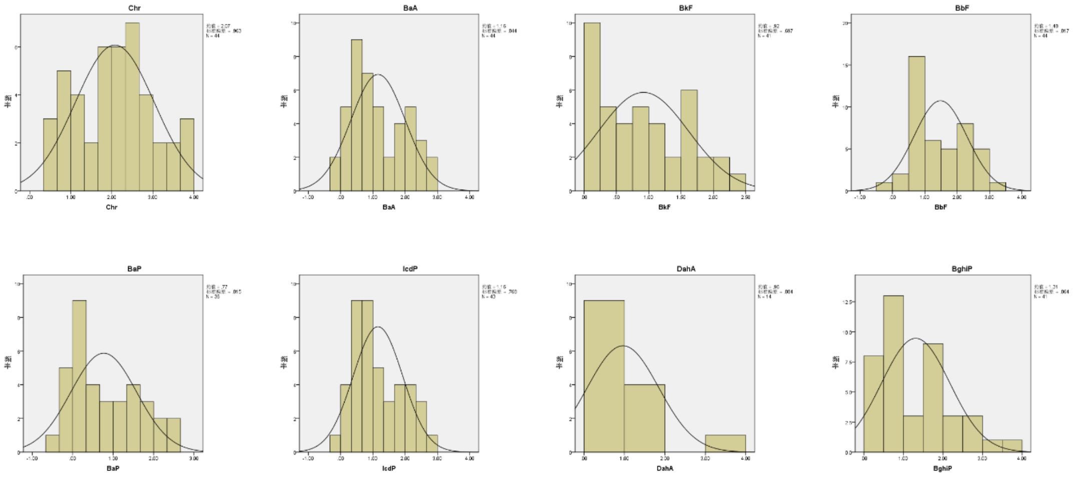

after (b)

Figure S3. Frequency distribution histogram of PAHs in plants before (a) and after (b) logarithmic transformation.

Table S4. Skewness, kurtosis and normal distribution test results of PAHs in plants before and after logarithmic transformation.

PAHs	Before			After(ln)		
	Skewness	Kurtosis	p	Skewness	Kurtosis	p
Nap	0.893	0.763	0.038	0.144	-0.048	0.952
Ace	0.504	-0.594	0.061	0.100	-0.825	0.355
Any	3.984	19.816	0.000	1.486	2.595	0.000
Flu	1.362	1.382	0.000	0.735	-0.008	0.026
Phe	1.325	1.809	0.001	0.453	0.115	0.452
Ant	1.585	3.985	0.000	0.385	0.255	0.837
Fla	3.412	12.255	0.000	1.087	1.326	0.005
Pyr	2.973	10.325	0.000	0.882	0.580	0.024
Chr	1.744	2.383	0.000	0.085	-0.745	0.297
BaA	1.664	2.319	0.000	0.510	-0.893	0.012
BkF	1.175	0.781	0.000	0.384	-1.177	0.005
BbF	1.336	0.835	0.000	0.323	-0.922	0.043
BaP	1.730	2.850	0.000	0.457	-0.870	0.078
IcdP	1.955	4.566	0.000	0.594	-0.576	0.035
DahA	6.020	38.246	0.000	2.068	5.438	0.005
BghiP	3.503	15.445	0.000	0.792	0.058	0.014

Table S5. Contents of PAHs in soil near each lake (ng/g).

	Chr	BaA	Pyr	BbF	BkF	BaP	IcdP	DahA	BghiP	Nap	Any	Ace	Flu	Phe	Ant	Fla
Chaohu	6.95	5.55	12.45	4.69	7.44	3.47	4.65	1.18	6.05	10.90	0.50	1.30	6.86	39.69	4.59	14.36
Danjiangkou Reservoir	13.07	10.01	20.40	8.81	13.99	6.30	8.99	1.65	9.05	11.74	1.20	1.66	7.41	45.98	5.22	24.09
Hongze Lake	3.56	2.33	6.79	2.95	4.68	1.81	2.92	0.53	3.72	17.52	0.39	1.73	9.36	50.88	5.31	9.13
Longgan Lake	3.55	1.60	5.74	1.33	2.11	0.81	1.44	ND [1]	1.46	23.13	ND [1]	1.71	11.45	55.95	4.63	9.57
Luoma Lake	2.27	1.38	4.49	1.56	2.48	0.52	1.19	0.34	1.48	14.79	ND [1]	1.62	8.11	48.33	4.06	6.56
Qiandao Lake	58.74	42.22	74.34	31.64	50.23	40.63	35.01	7.15	42.04	20.76	2.00	3.25	7.22	52.49	6.89	77.52
Wuhan East Lake	33.55	20.37	36.47	22.67	35.99	23.01	26.08	5.28	30.22	21.95	4.46	2.44	10.75	67.60	7.90	46.18
Changhu	11.62	11.01	21.82	9.61	15.25	9.60	11.07	2.15	11.50	14.22	0.99	1.54	9.215	57.75	7.50	25.85
Liangzi Lake	13.06	9.10	16.17	5.25	8.33	4.11	7.75	1.42	7.38	25.27	ND [1]	1.79	7.89	47.28	3.96	21.78

[1] ND means not detected

Table S6. Contents of PAHs in plants near each lake (ng/g).

Lake	Plant	Chr	BaA	Pyr	BbF	BkF	BaP	IcdP	DahA
Hongze Lake (1-8)	1-*Pisum sativum* L.	2.40	0.99	8.79	2.16	1.29	0.77	1.85	ND [1]
	2-*Setaria viridis* (L.) Beauv.	7.69	2.70	8.01	4.49	2.06	0.73	1.60	ND [1]
	3-Populus	9.26	2.76	10.87	3.42	2.24	ND [1]	1.60	ND [1]
	4-*Erigeron* L.	6.17	1.72	12.41	2.54	1.18	1.02	1.63	ND [1]
	5-*Trifolium* L.	6.69	3.05	12.13	3.41	1.98	1.31	2.48	1.04
	6-*Achyranthes bidentata* Blume	11.20	2.41	10.29	3.84	2.22	1.33	2.29	ND [1]
	7-*Morus alba* L.	12.40	4.13	14.71	7.62	5.11	2.02	3.62	ND [1]
	8-*Bidens pilosa* L.	41.58	11.11	40.82	11.60	5.06	3.12	4.67	ND [1]
Qiandao Lake (9-12)	9-*Cunninghamia lanceolata* (Lamb.) Hook.	1.67	0.98	3.71	1.11	ND [1]	ND [1]	1.00	ND [1]
	10-Compositae	4.52	1.24	6.27	1.86	ND [1]	ND [1]	ND [1]	ND [1]
	11-*Saccharum* L.	1.52	1.04	6.19	0.95	ND [1]	ND [1]	1.06	1.13
	12-*Lindera* Thunb.	3.21	1.82	8.14	2.15	1.29	1.44	1.62	34.64
Changhu (13-15)	13-*Cynodon dactylon* (L.) Pers.	5.50	3.03	7.81	2.66	1.67	1.08	2.79	ND [1]
	14-*Alternanthera sessilis* (L.) DC.	8.80	1.54	4.84	2.72	1.33	1.02	1.73	ND [1]
	15-*Setaria viridis* (L.) Beauv.	6.74	2.68	9.32	3.54	2.23	1.38	3.28	ND [1]
Wuhan East Lake (16-20)	16- *Celtis* L.	47.18	9.39	25.21	18.80	8.43	7.41	11.42	1.48
	17-*Sedum aizoon* L.	26.02	6.34	29.39	8.98	5.60	3.14	6.82	ND [1]
	18-*Hypericum* Linn	37.84	11.54	66.83	15.69	7.20	3.80	7.80	ND [1]

	19-*Imperata cylindrica* (L.) Beauv.	22.62	13.52	34.88	18.36	10.20	11.67	19.96	3.62
	20-*Scirpus validus* Vahl	13.67	5.08	8.49	10.66	5.53	4.12	9.83	1.61
Chaohu (21-26)	21-*Zea mays* L.	5.15	2.39	10.64	4.23	2.03	1.83	3.35	ND [1]
	22-*Vigna radiata* (Linn.) Wilczek	15.06	9.45	18.41	11.61	6.94	8.05	11.98	2.53
	23-*Setaria viridis* (L.) Beauv.	2.91	1.59	5.15	2.65	1.22	1.35	2.60	ND [1]
	24-*Gaillardia pulchella* Foug.	10.36	6.37	23.00	7.82	4.24	5.46	6.79	2.94
	25-*Koelreuteria paniculata* Laxm.	19.23	7.43	15.09	12.99	5.33	5.75	9.26	1.82
	26-*Allium ascalonicum* L.	8.79	3.03	9.78	4.60	2.52	2.00	3.95	6.04
Liangzi Lake (27-31)	27- *Pennisetum alopecuroides* (L.) Spreng.	1.98	1.09	3.55	1.58	1.04	0.59	1.82	ND [1]
	28-*Miscanthus sinensis* Anderss.	3.06	1.50	7.10	2.29	1.14	0.82	1.99	ND [1]
	29-*Bidens frondosa* L.	11.18	2.77	12.64	2.54	1.24	ND [1]	2.01	ND [1]
	30-Arthraxon Beauv	3.42	1.85	5.28	2.56	1.30	0.99	2.32	ND [1]
	31-*Polygonum hydropiper* L.	7.03	2.20	9.39	2.43	1.27	0.98	2.42	ND [1]
Danjiangkou Reservoir (32-36)	32-*Setaria viridis* (L.) Beauv.	35.39	18.19	57.68	20.85	7.72	10.33	12.83	3.44
	33-*Erigeron annuus* (L.) Pers.	49.23	17.78	104.13	15.92	5.55	5.72	7.62	2.46

	34-*Miscanthus sinensis* Anderss.	18.15	9.19	31.62	8.50	3.44	3.86	5.69	1.71
	35-*Atractylodes Lancea* (Thunb.) DC.	13.91	7.46	25.68	9.69	3.75	4.45	5.46	1.76
	36-*Cynodon dactylon* (L.) Pers.	11.44	6.33	19.58	6.68	2.85	3.51	5.05	ND [1]
Longgan Lake (37-41)	37-*Melia azedarach* L.	15.90	2.00	15.94	7.17	3.45	1.59	1.84	ND [1]
	38-*Acalypha australis* L.	5.37	1.26	5.97	1.91	1.24	ND [1]	1.22	ND [1]
	39-*Digitaria sanguinalis* (L.) Scop.	2.61	1.51	8.24	1.92	1.03	ND [1]	1.26	ND [1]
	40-*Zea mays* L.	2.08	1.48	8.31	2.49	1.08	ND [1]	1.16	ND [1]
	41-*Rostellularia procumbens* (L.) Nees.	9.80	2.28	11.00	3.94	3.02	1.57	2.37	ND [1]
Luoma Lake (42-44)	42-*Phragmites australis* (Cav.) Trin. ex Steud.	2.56	1.87	8.54	2.17	1.49	1.17	2.23	ND [1]
	43-*Cosmos bipinnata* Cav.	1.64	1.39	8.61	2.17	1.15	1.18	1.85	ND [1]
	44-*Desmodium microphyllum* (Thunb.) DC.	9.29	5.65	10.88	5.05	2.71	2.47	3.12	ND [1]

		BghiP	Nap	Any	Ace	Flu	Phe	Ant	Fla
Hongze Lake (1-8)	1-*Pisum sativum* L.	1.56	29.36	1.05	4.37	21.93	93.50	9.01	15.70
	2-*Setaria viridis* (L.) Beauv.	1.54	26.78	0	3.00	19.78	90.18	8.59	14.20
	3-Populus	1.28	28.57	1.14	4.02	21.98	88.33	10.13	19.56
	4-*Erigeron* L.	1.77	44.66	2.64	4.82	28.61	128.03	10.45	25.96
	5-*Trifolium* L.	2.28	25.57	0.82	3.40	23.36	114.10	9.82	21.67

	6-*Achyranthes bidentata* Blume	1.78	31.16	0.89	4.24	23.17	103.09	11.29	17.94
	7-*Morus alba* L.	2.88	32.19	1.26	4.51	25.27	110.16	10.07	24.35
	8-*Bidens pilosa* L.	4.55	34.53	1.25	5.83	25.69	142.18	9.37	54.88
Qiandao Lake (9-12)	9-*Cunninghamia lanceolata* (Lamb.) Hook.	ND [1]	34.03	2.04	4.37	17.98	64.08	5.39	8.89
	10-Compositae	ND [1]	39.04	ND [1]	3.87	17.77	68.87	5.59	12.10
	11-*Saccharum* L.	ND [1]	34.67	1.03	3.65	19.11	91.05	6.84	11.48
	12-*Lindera* Thunb.	1.38	40.91	5.37	6.53	47.20	115.23	8.83	18.51
Changhu (13-15)	13-*Cynodon dactylon* (L.) Pers.	1.94	30.42	0.93	2.86	17.40	71.16	6.13	12.71
	14-*Alternanthera sessilis* (L.) DC.	1.56	25.39	1.87	2.65	14.33	57.08	5.09	7.39
	15-*Setaria viridis* (L.) Beauv.	5.25	32.58	1.11	2.90	16.27	74.58	6.97	13.90
Wuhan East Lake (16-20)	16-*Celtis* L.	10.51	32.21	2.32	4.83	23.32	112.77	11.10	59.64
	17-*Sedum aizoon* L.	6.10	41.82	1.85	5.58	33.45	137.06	14.52	44.23
	18-*Hypericum* Linn	5.59	28.76	6.34	4.53	32.20	214.98	8.67	207.91
	19-*Imperata cylindrica* (L.) Beauv.	20.89	56.72	3.05	6.59	44.08	190.06	20.84	49.64
	20-*Scirpus validus* Vahl	6.32	23.96	2.80	3.73	19.93	77.53	7.73	17.57
Chaohu (21-26)	21-*Zea mays* L.	3.56	25.09	3.07	3.75	20.45	105.94	6.355	24.21
	22-*Vigna radiata* (Linn.) Wilczek	12.70	22.43	1.57	3.72	21.23	110.05	6.77	34.14
	23-*Setaria viridis* (L.) Beauv.	2.43	26.55	1.16	3.21	19.35	70.92	4.91	11.95

	24-*Gaillardia pulchella* Foug.	7.28	25.61	14.68	3.70	47.76	185.12	7.96	53.57
	25-*Koelreuteria paniculata* Laxm.	8.29	30.54	2.10	3.90	23.06	108.21	8.38	32.73
	26-*Allium ascalonicum* L.	4.31	30.94	1.01	4.30	32.02	148.34	6.32	25.58
Liangzi Lake (27-31)	27-*Pennisetum alopecuroides* (L.) Spreng.	1.61	17.20	ND [1]	3.04	15.95	61.09	4.94	7.45
	28-*Miscanthus sinensis* Anderss.	2.10	19.24	1.65	5.45	33.91	116.13	8.61	15.24
	29-*Bidens frondosa* L.	2.43	23.49	1.17	4.58	28.72	110.67	6.87	27.43
	30-Arthraxon Beauv	13.89	20.30	1.06	3.20	19.85	77.78	6.46	11.42
	31-*Polygonum hydropiper* L.	2.16	28.29	2.91	2.92	15.41	99.95	5.99	19.28
Danjiangkou Reservoir (32-36)	32-*Setaria viridis* (L.) Beauv.	14.82	61.44	2.91	5.67	27.76	156.68	12.36	93.67
	33-*Erigeron annuus* (L.) Pers.	8.57	42.52	4.95	5.68	27.80	226.42	10.95	208.17
	34-*Miscanthus sinensis* Anderss.	6.08	31.25	1.80	5.58	22.38	108.05	8.36	55.01
	35-*Atractylodes Lancea* (Thunb.) DC.	6.01	32.48	1.50	3.47	18.37	89.04	7.11	51.00
	36-*Cynodon dactylon* (L.) Pers.	5.41	37.44	1.18	3.59	18.46	96.17	7.94	33.55
Longgan Lake (37-41)	37-*Melia azedarach* L.	1.52	35.90	1.21	4.50	22.86	88.61	7.67	23.80
	38-*Acalypha australis* L.	1.06	29.00	ND [1]	3.68	21.45	87.85	9.14	9.45

	39-*Digitaria sanguinalis* (L.) Scop.	1.87	45.26	1.31	5.97	31.97	128.65	11.03	15.33
	40-*Zea mays* L.	41.20	45.94	1.21	5.66	45.04	142.47	15.61	13.34
	41-*Rostellularia procumbens* (L.) Nees.	2.26	41.63	1.19	4.79	30.77	122.15	12.17	18.41
Luoma Lake (42-44)	42-*Phragmites australis* (Cav.) Trin. ex Steud.	2.49	47.36	1.27	4.68	23.22	97.68	5.61	13.83
	43-*Cosmos bipinnata* Cav.	2.25	50.05	1.70	4.00	18.59	85.14	4.06	16.23
	44-*Desmodium microphyllum* (Thunb.) DC.	2.55	30.23	1.27	3.49	18.13	89.28	5.05	21.87

[1] ND means not detected

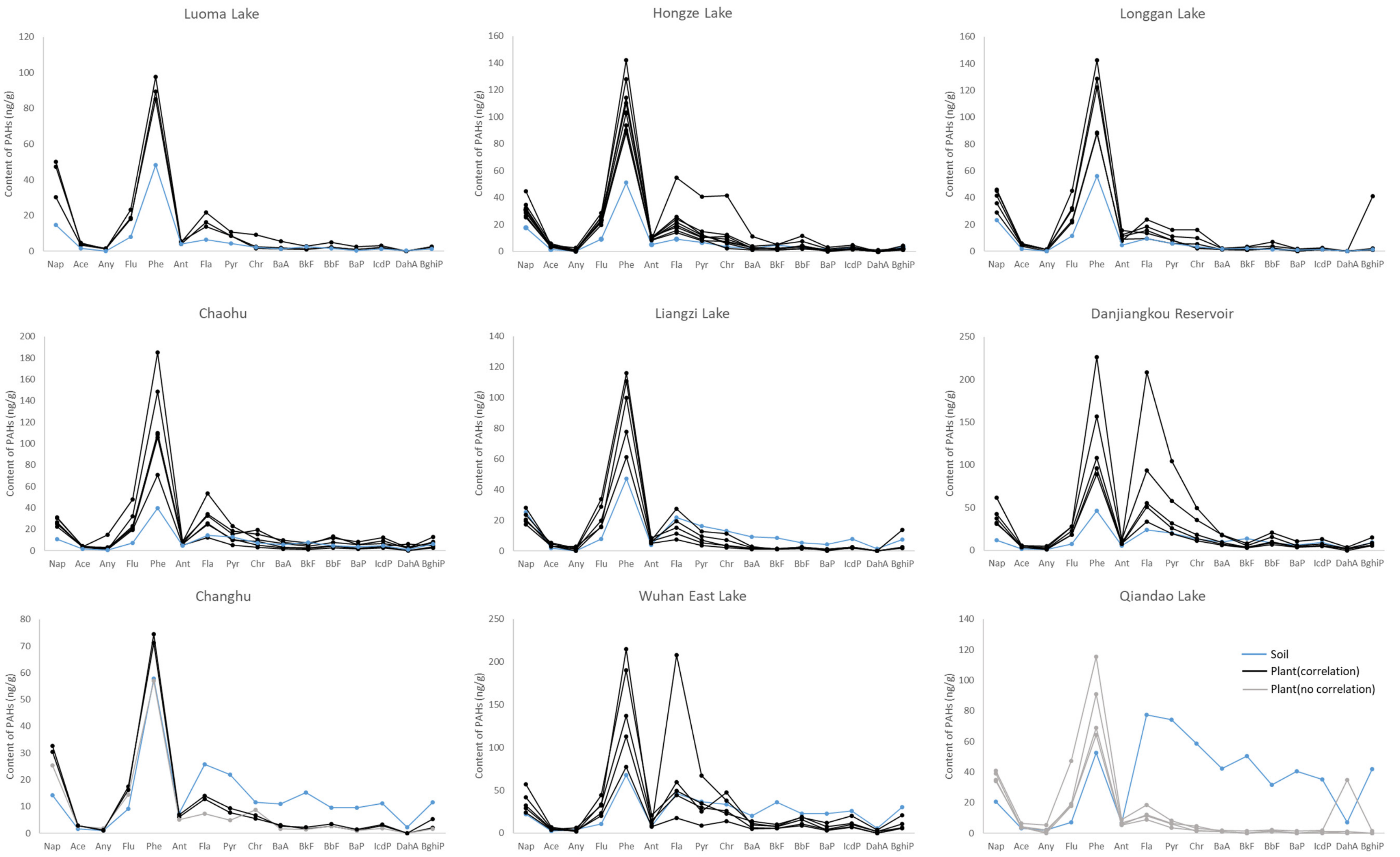

Figure S4. Contents of PAH monomers in soil and plants near different lakes.

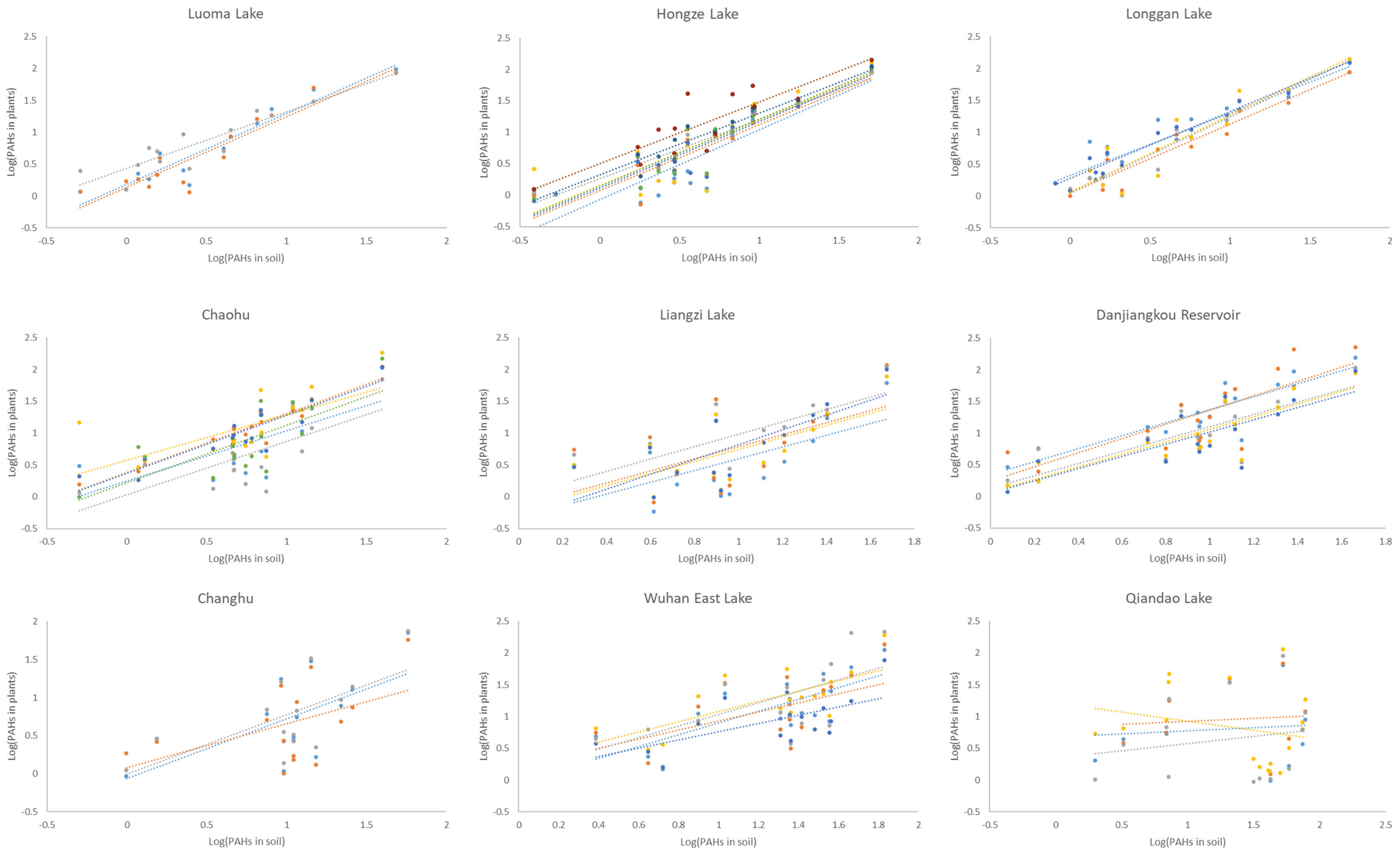

Figure S5. Correlation of PAH contents in soil and plants near different lakes.

Table S7. Correlation coefficient, p, linear fitting function and R^2 of PAH monomer contents in soil and plants in different lake areas.

Lake	Plant	Correlation coefficent	p	Linear fitting function	R^2
Hongze Lake (1-8)	1-*Pisum sativum* L.	0.844	0.000	y = 1.1101x-0.0647	0.7313
	2-*Setaria viridis* (L.) Beauv.	0.879	0.000	y=1.0444x+0.0746	0.8075
	3-Populus	0.857	0.000	y=0.9403x+0.2669	0.8496
	4-*Erigeron* L.	0.771	0.000	y=1.0396x+0.1542	0.6505
	5-*Trifolium* L.	0.868	0.000	y=1.0512x+0.1181	0.8499
	6-*Achyranthes bidentata* Blume	0.841	0.000	y=1.0623x+0.1431	0.8366
	7-*Morus alba* L.	0.906	0.000	y=0.9795x+0.3262	0.903
	8-*Bidens pilosa* L.	0.774	0.000	y=0.9769x+0.503	0.7591
Qiandao Lake (9-12)	9-*Cunninghamia lanceolata* (Lamb.) Hook.	-0.034	0.900	y=0.0952x+0.6795	0.0094
	10-Compositae	0.236	0.380	y=0.0918x+0.8336	0.0078
	11-*Saccharum* L.	0.047	0.862	y=0.2236x+0.3525	0.0326
	12-*Lindera* Thunb.	-0.147	0.587	y=-0.2881x+1.2169	0.0523
Changhu (13-15)	13-*Cynodon dactylon* (L.) Pers.	0.544	0.029	y=0.7819x-0.0578	0.4103
	14-*Alternanthera sessilis* (L.) DC.	0.391	0.134	y=0.5732x+0.0845	0.2451
	15-*Setaria viridis* (L.) Beauv.	0.597	0.015	y=0.7811x-0.0042	0.4464
Wuhan East Lake	16- *Celtis* L.	0.685	0.003	y=0.931x-0.0338	0.5697
	17-*Sedum*	0.562	0.024	y=0.7147x+0.2168	0.3035

Lake	Plant	Correlation coefficent	p	Linear fitting function	R^2
(16-20)	*aizoon* L.				
	18-*Hypericum* Linn	0.606	0.013	y=0.9067x+0.1275	0.4059
	19-*Imperata cylindrica* (L.) Beauv.	0.606	0.013	y=0.7995x+0.2799	0.502
	20-*Scirpus validus* Vahl	0.562	0.024	y=0.648x+0.1159	0.4103
Chaohu (21-26)	21-*Zea mays* L.	0.697	0.003	y=0.7907x+0.2491	0.486
	22-*Vigna radiata* (Linn.) Wilczek	0.882	0.000	y=0.9249x+0.3832	0.9084
	23-*Setaria viridis* (L.) Beauv.	0.676	0.004	y=0.8393x+0.0347	0.4825
	24-*Gaillardia pulchella* Foug.	0.656	0.006	y=0.711x+0.5763	0.4392
	25-*Koelreuteria paniculata* Laxm.	0.821	0.000	y=0.9104x+0.3715	0.8209
	26-*Allium ascalonicum* L.	0.671	0.004	y=0.9029x+0.2199	0.569
Liangzi Lake (27-31)	27-*Pennisetum alopecuroides* (L.) Spreng.	0.664	0.005	y=0.9222x-0.3236	0.3753
	28-*Miscanthus sinensis* Anderss.	0.565	0.023	y=0.946x-0.1565	0.3278
	29-*Bidens frondosa* L.	0.726	0.001	y=0.9742x+0.0088	0.4655
	30-Arthraxon Beauv	0.635	0.008	y=0.9435x-0.2004	0.4368
	31-*Polygonum hydropiper* L.	0.635	0.008	y=1.1631x-0.3429	0.5798

Lake	Plant	Correlation coefficent	p	Linear fitting function	R^2
Danjiangkou Reservoir (32-36)	32-*Setaria viridis* (L.) Beauv.	0.806	0.000	y=1.0253x+0.344	0.7898
	33-*Erigeron annuus* (L.) Pers.	0.768	0.001	y=1.1248x+0.2409	0.6515
	34-*Miscanthus sinensis* Anderss.	0.741	0.001	y=0.9692x+0.1305	0.6511
	35-*Atractylodes Lancea* (Thunb.) DC.	0.762	0.001	y=0.9945x+0.0644	0.7198
	36-*Cynodon dactylon* (L.) Pers.	0.685	0.003	y=0.9628x+0.0555	0.6134
Longgan Lake (37-41)	37-*Melia azedarach* L.	0.935	0.000	y=0.9792x+0.322	0.8791
	38-*Acalypha australis* L.	0.951	0.000	y=1.0822x+0.0456	0.9255
	39-*Digitaria sanguinalis* (L.) Scop.	0.887	0.000	y=1.1824x+0.0599	0.8862
	40-*Zea mays* L.	0.799	0.000	y=1.204x+0.0679	0.8537
	41-*Rostellularia procumbens* (L.) Nees.	0.952	0.000	y=1.0526x+0.2737	0.9567
Luoma Lake (42-44)	42-*Phragmites australis* (Cav.) Trin. ex Steud.	0.912	0.000	y=1.1155x+0.1789	0.9081
	43-*Cosmos bipinnata* Cav.	0.809	0.000	y=1.1111x+0.1346	0.8649
	44-*Desmodium microphyllum* (Thunb.) DC.	0.889	0.000	y=0.8871x+0.4296	0.8516
